# Inhibition of Xanthine Oxidase by Four Phenolic Acids: Kinetic, Spectroscopic, Molecular Simulation, and Cellular Insights

**DOI:** 10.3390/foods14193404

**Published:** 2025-10-01

**Authors:** Xiao Wang, Di Su, Xinyu Luo, Bingjie Chen, Khushwant S. Bhullar, Hongru Liu, Chunfang Wang, Jinglin Zhang, Longshen Wang, Hang Yang, Wenzong Zhou

**Affiliations:** 1 Crop Breeding and Cultivation Research Institution, Research Center for Agricultural Products Preservation and Processing, Shanghai Academy of Agricultural Sciences, Shanghai 201403, China; wangxiao.0127@163.com (X.W.); 212303817025@zust.edu.cn (X.L.); chenbingjie0204@126.com (B.C.); 20200203@saas.sh.cn (H.L.); fhwcf@126.com (C.W.); 2020208034@stu.njau.edu.cn (J.Z.); wlsh@saas.sh.cn (L.W.); 2 Teaching Experimental Center for Pharmacy, School of Pharmaceutical Sciences, Shanghai Jiao Tong University, 800 Dongchuan Road, Shanghai 200240, China; sudi@sjtu.edu.cn; 3 Department of Agricultural Food & Nutritional Science, University of Alberta, Edmonton, AB T6G 2P5, Canada; bhullar@ualberta.ca; 4 Key Laboratory of Integrated Rice-Fish Farming Ecosystem, Ministry of Agriculture and Rural Affairs, Shanghai Academy of Agricultural Sciences, Shanghai 201403, China

**Keywords:** phenolic acids, XOD inhibition, molecular docking, molecular dynamics simulation, cell models, ADMET analysis

## Abstract

The inhibition mechanism and binding properties of four phenolic acids (ferulic acid (FA), *p*-coumaric acid (CA), gallic acid (GA), and protocatechuic acid (PA)) on xanthine oxidase (XOD) were investigated. All four phenolic acids acted via a mixed inhibition pattern, mainly influencing the hydrophobic regions and secondary conformation of XOD through hydrophobic bonding and hydrophobic association. Molecular dynamics simulations exhibited that the complexes of XOD with FA and CA revealed smaller radii of gyration (Rg) and solvent-accessible surface areas (SASA), along with lower variability in root-mean-square deviation (RMSD) and root-mean-square fluctuation (RMSF), collectively indicating greater structural stability. FA, CA, and PA significantly reduced uric acid (UA) concentration in the 25–100 μM range. Although GA only reduced UA levels in cell models at 25 μM, this effect was likely due to its larger polar surface area, which limits cellular uptake. Absorption, distribution, metabolism, excretion, and toxicity (ADMET) evaluation suggested that these phenolic acids have potential for development.

## 1. Introduction

Hyperuricemia is a metabolic condition associated with unususlly high concentrations of uric acid (UA) in blood [1]. It is often associated with various pathological conditions, including gout, arthritis, and renal and cardiovascular diseases [2]. Xanthine oxidase (XOD) plays a central role in hyperuricemia by catalyzing the oxidation of hypoxanthine to xanthine, which is subsequently converted to UA, along with the production of superoxide anion and hydrogen peroxide (H_2_O_2_) [3]. The excessive generation of reactive oxygen species (ROS) during UA formation has been involved in the onset of vascular disease, hypertension, aging, and diabetes. Several synthetic drugs, such as allopurinol and febuxostat, are currently applied in clinical practice to manage hyperuricemia and gout [4]. However, these drugs are associated with side effects, including skin rashes, nephrotoxicity, and gastrointestinal discomfort [5]. Therefore, the discovery of natural compounds with potent XOD inhibitory and antioxidant activities holds significant therapeutic potential for hyperuricemia and related oxidative stress disorders [6,7].

Rice bran is an abundant source of various biologically active components, particularly phenolic acids, which belong to the polyphenol family [8]. The major phenolic acids detected in rice bran include ferulic acid (FA), *p*-coumaric acid (CA), gallic acid (GA), and protocatechuic acid (PA) [9,10]. These phenolic acids display a broad spectrum of health benefits, encompassing antioxidant, hepatoprotective, anti-inflammatory, antibacterial, and anticancer activities. Furthermore, these phenolic acids demonstrate significant therapeutic potential as inhibitors of XOD, highlighting their importance in hyperuricemia research [11]. Beyond rice bran, phenolic acids are abundant in numerous dietary sources, such as grains, vegetables, and fruits [12,13]. Recent investigations have revealed the importance of phenolic acids in reducing serum UA levels and alleviating metabolic diseases such as gout. For instance, Lou et al. demonstrated that FA, a mixed-type inhibitor, significantly inhibits UA production in hyperuricemic cell models by suppressing XOD activity [14]. Furthermore, Li et al. proposed that both FA and CA can enhance UA excretion through the stimulation of the PI3K/Akt signaling pathway, thereby mitigating the risk of hyperuricemia [15]. Yang et al. further observed that CA and its derivatives inhibit XOD activity, thereby reducing UA production [16]. Additionally, Wu et al. also explored the major bioactive constituents in different teas, finding that GA exhibited the most pronounced inhibitory effect on UA production, followed by tea polyphenols and theaflavins [17]. Despite these promising inhibitory effects, the precise mechanisms underlying the interaction of phenolic acids with XOD and the resulting differences in inhibitory activity remain poorly understood. This highlights the need for further investigation into the structure-activity relationships of phenolic acids to clarify their interaction mechanisms and enhance their application as XOD inhibitors.

The principal aim of this study is to further investigate the mechanisms by which rice bran-derived phenolic acids inhibit XOD activity. The inhibitory effects of four major rice bran phenolic acids (FA, CA, GA, and PA) on XOD were evaluated via in vitro enzyme inhibition kinetics. To elucidate the interaction mechanisms, fluorescence spectroscopy, circular dichroism (CD), molecular docking and dynamics simulations were employed to analyze the interactions between phenolic acids and XOD and their effects on enzyme structure. Additionally, the metabolic kinetics and potential toxicity of these phenolic acids in the human body were assessed. The structure-activity relationships of these phenolic acids in XOD inhibition were also explored. The findings reveal significant information about the mechanisms underlying phenolic acid-mediated XOD inhibition.

## 2. Materials and Methods

### 2.1. Materials

Xanthine, XOD (7.2 units/mL, derived from bovine milk), allopurinol, FA, CA, GA, PA, and UA (purity > 98%) were obtained from Sigma-Aldrich (St. Louis, MO, USA). 6-Hydroxy-2,5,7,8-tetramethylchromane-2-carboxylic acid (Trolox), 2,2-diphenyl-1-picrylhydrazyl (DPPH) and 2,2′-azobis(2-methylpropionamidine) dihydrochloride (AAPH) were supplied by Beijing Essan Huitong Technology Co., Ltd. (Beijing, China). 2,2′-Azinobis(3-ethylbenzothiazoline-6-sulfonic acid) diammonium salt (ABTS) and sodium fluorescein were acquired from Beijing Zhongcheng Honnin Science and Technology Co., Ltd. (Beijing, China). HPLC-grade solvents used for high-performance liquid chromatography analysis were purchased from Sigma-Aldrich. BRL-3a cells were obtained from Wuhan Purcell Life Sciences Co., Ltd. (Wuhan, China). The UA assay kit (C012-2-1) was procured from Nanjing Jiancheng Institute (Nanjing, China). Minimum essential medium (MEM), fetal bovine serum (FBS), and penicillin/streptomycin were purchased from Life Technologies (Carlsbad, CA, USA). All remaining reagents were of analytical grade.

### 2.2. Assessment of Antioxidant Activity

#### 2.2.1. Evaluation of Reducing Power

The reducing capacity was assessed following the method of Wang et al., with appropriate alterations [18]. Briefly, a 0.5 mL of sample at final concentrations of 0.125, 0.25, 0.5, 1.0, and 2.0 mg/mL was combined with 0.5 mL of 0.2 M phosphate buffer (PBS, pH 6.6) and 0.5 mL of 1% (,*w*/*v*) potassium ferricyanide solution. The mixture was incubated at 50 °C for 20 min, after which 0.5 mL of 10% (,*w*/*v*) trichloroacetic acid solution was added. Following centrifugation at 3000 rpm for 10 min, 0.5 mL of the supernatant was transferred to a new test tube, and 0.5 mL of distilled water along with 0.1 mL of 0.1% (,*w*/*v*) ferric chloride solution were added. After 10 min of incubation, absorbance was measured at 700 nm. Higher absorbance values correspond to stronger reducing activity. The IC_50_ value was defined as the concentration at which the absorbance reached 0.5.

#### 2.2.2. Measurement of ABTS Radical Scavenging Activity

The modified ABTS assay was carried out as described by Foss et al. with appropriate modifications [19]. A 7 mM ABTS solution was mixed in equal volumes with 2.45 mM potassium persulfate and kept in the dark at room temperature for 12–16 h to generate the ABTS•^+^ stock solution. Before application, the stock was diluted with 10 mM PBS (pH 7.4) and adjusted to an absorbance of 0.700 ± 0.020 at 734 nm. Then, 250 μL of the diluted ABTS•^+^ solution was mixed with 10 μL of the sample, vortexed for 30 s, and incubated at 30 °C for 30 min. Absorbance was recorded at 734 nm. PBS served as the blank, while Trolox was used as the standard. The results were reported as IC_50_ value, representing the concentration of sample required to scavenge 50% of ABTS•^+^ radicals.

#### 2.2.3. Determination of DPPH Radical Scavenging Activity

The DPPH• scavenging capacity was evaluated following the method of Nakbi et al. [20]. A 5.0 mg/mL stock solution was prepared in anhydrous ethanol and subsequently diluted to final concentrations of 60, 120, 180, 240, and 300 μg/mL. In a 96-well plate, 240 μL of 0.2 mM DPPH solution was combined with 10 μL of each sample solution. The mixture was vortexed and incubated at room temperature for 30 min. Absorbance was then measured at 517 nm and recorded as Ai. For the control experiment, 10 μL of anhydrous ethanol replaced the sample soluition, while all other steps were identical, and the absorbance was recorded as A_c_. To correct for background, 10 μL of sample solution was mixed with 240 μL of anhydrous ethanol alone, and the absorbance at 517 nm was recorded as *A*_0_. The results were expressed as IC_50_.

(1) $$\mathrm{Inhibition}\;(\%)=\frac{Ac-(Ai-A0)}{Ac}\times 100\%$$

#### 2.2.4. Evaluation of Oxygen Radical Absorption Capacity (ORAC)

The ORAC analysis method was adapted from Wang et al.with adjustments [21]. The trial was conducted in 75 mM PBS (pH 7.4). A 39.9 μM sodium fluorescein stock solution was prepared, kept at 4 °C in under dark conditions, and then adjusted to 159 nM for use. AAPH was freshly dissolved in PBS at 38.25 mM and maintained in an ice-water bath. In a 96-well microplate, 25 μL of the sample solution or buffer (blank) was mixed with 75 μL of 159 nM sodium fluorescein, followed by incubation at 37 °C for 15 min. The reaction was initiated by adding 100 μL of AAPH solution, and fluorescence intensity was recorded every minute for 100 min to generate a fluorescence decay curve (Thermo Fisher Scientific, Waltham, MA, USA). Trolox served as the reference standard, while PBS functioned as the blank. ORAC values were determined from the net area under the fluorescence decay curve and expressed as Trolox equivalents per sample [22].

### 2.3. Determination of XOD Inhibitory Activity In Vitro

The XOD inhibitory activity of the samples was assessed using an enzymatic reaction followed by HPLC analysis [23]. Briefly, 50 μL of XOD (0.05 U/mL in 200 mM PBS, pH 7.5) was combined with either 50 μL of PBS (control) or 50 μL of the sample solution. The reaction mixture was equilibrated at 37 °C for 5 min, after which 150 μL of 0.40 mM xanthine was added. The reaction proceeded at 37 °C for 10 min and was terminated by adding 80 μL of 1.0 M HCl.

UA production was measured using an HPLC system (Agilent Technologies, Santa Clara, CA, USA) equipped with a UV detector. The reaction solution was diluted 20-fold and filtered, and 10 μL of the filtrate was injected into an Agilent Eclipse XDB-C18 column (150 × 4.6 mm, 5 μm). The mobile phase, consisting of 10% methanol and 90% 10 mM sodium phosphate buffer (pH 5.5), was delivered isocratically at 1.0 mL/min. UA was detected at 292 nm with the column maintained at 25 °C. Concentrations were calculated based on an external calibration curve. XOD inhibition was expressed as the percentage reduction of UA formation, and the IC_50_ value was defined as the sample concentration at which 50% inhibition was achieved.

### 2.4. Kinetic Mechanisms of XOD Inhibition

The inhibition kinetics of XOD by FA, CA, GA, and PA were evaluated using Lineweaver–Burk plots [24]. The inhibitor concentrations were as follows: FA (0, 2.0, 3.0, and 4.0 mM), CA (0, 1.0, 2.0, and 4.0 mM), GA (0.2, 1.0, 2.0, and 4.0 mM), and PA (1.0, 2.0, 5.0, and 10.0 mM). The substrate (xanthine) concentrations were fixed at 0.1, 0.2, 0.3, and 0.4 mM. XOD (0.05 U/mL) was used, and the initial reaction velocity (v) was calculated based on UA formation.

Lineweaver–Burk plots were established by plotting 1/v against 1/[s] for each inhibitor concentration. Four linear regressions were obtained for each inhibitor, enabling the determination of the inhibition type. In the case of mixed inhibition, the Lineweaver–Burk equation is given by: (2). The slope and intercept of the Lineweaver–Burk plot were plotted against inhibitor concentration [I], yielding the following equations: (3) and (4).

(2) $$\frac{1}{v}=\frac{K_{m}}{v_{\max}}\frac{1}{\left[s\right]}+\frac{1}{v_{\max}}(1+\frac{\left[I\right]}{K_{i}})*(1+\frac{\left[I\right]}{K_{\mathrm{is}}}),$$

(3) $$Slope=\frac{K_{m}}{v_{max}}+\frac{K_{m}\left[I\right]}{v_{\max}K_{i}},$$

(4) $$Y_{\mathrm{intercept}}=\frac{1}{v_{\max}}+\frac{\left[I\right]}{v_{\max}K_{\mathrm{is}}},$$

V denotes the reaction rate, V_max_ the maximum rate, K_m_ the Michaelis constant, K_i_ the inhibition constant for XOD, and K_is_ the inhibition constant for the XOD–substrate complex. Decreased K_i_ or K_is_ values indicate stronger inhibitory activity.

### 2.5. Fluorescence Spectra Measurement

The binding interaction between XOD and the phenolic compounds FA, CA, GA, and PA was investigated using a fluorescence quenching assay, following the method of Liu et al. [25]. In brief, 100 µL of XOD (0.05 U/mL in 200 mM PBS, pH 7.5) was combined with 100 µL of each phenolic acid at final concentrations of 0, 50, 100, 200, 300, 400, and 500 μM. The mixtures were incubated at 25 °C for 10 min, and fluorescence emission spectra were recorded using a microplate reader (Thermo Fisher Scientific, Waltham, MA, USA) with an excitation wavelength of 280 nm and an emission collected from 310 to 500 nm. PBS was used as the blank, and background fluorescence was subtracted to ensure accuracy.

### 2.6. CD Spectroscopic Analysis

CD spectroscopy was employed to determine the conformational structure of XOD in the presence of phenolic compounds. Spectra were recorded in the wavelength range of 190–250 nm using a scanning rate of 100 nm/min, with continuous nitrogen purging at room temperature [26]. XOD was prepared at 2.0 mg/mL in PBS (200 mM, pH 7.5) and then diluted 6-fold to 0.33 mg/mL. Phenolic compounds were dissolved in PBS at 300 μM, and equal volumes of XOD and phenolic solutions were joined in equal amounts. CD spectra of the mixtures were recorded with the PBS as the background, and secondary structure content of XOD was analyzed employing the SELCON3 algorithm on the DichroWeb server (http://dichroweb.cryst.bbk.ac.uk/html/home.shtml).

### 2.7. Molecular Docking

The binding modes and interactions of FA, CA, GA, and PA with XOD were investigated through molecular docking analyses [27]. The crystal structure of bovine XOD (PDB code 3NVZ) complexed with indole-3-aldehyde was retrieved from the Protein Data Bank. Before docking, all water molecules and ligands were removed, and hydrogen atoms and charges were assigned with MGLTools 1.5.7. The three-dimensional structures of the phenolics were generated using ChewDraw 20.0. XOD and molecular docking of the phenolic acids were carried out using Autodock Tools 1.5.6, and the highest-scoring conjugates were considered the best docking results. Finally, the complexes were visualized using PyMol 2.5.0 and Discovery Studio 4.5.

### 2.8. Molecular Dynamics Simulations

Molecular dynamics simulations of phenolic acids and XOD were performed as described in previous reports [27]. During the pretreatment of phenolic acid molecules, Gaussian 16W was used for hydrogenation, and AmberTools 22 was employed to apply the GAFF force field. Amber99sb-ildn was used as the force field, and TIP3P water was served as the solvent model, with charge neutralization achieved by Na^+^ ions. Energy minimization was performed using the steepest descent method. Simulations were performed under an NPT ensemble at 300 K and 1 atm, with a coupling constant of 0.1 ns over a 100 ns production run. The conditions for the simulation were set to 300 K temperature, 1 atm pressure, and 100 ns in an isothermal–isobaric system. The root-mean-square deviation (RMSD), root-mean-square fluctuation (RMSF), hydrogen bond changes, radii of gyration (Rg), and solvent-accessible surface areas (SASA) were calculated from the molecular dynamics simulations [28].

### 2.9. Assessment of UA-Lowering Activity in BRL-3a Cells

#### 2.9.1. Cell Culture

BRL-3a cells were grown at 37 °C in a humidified 5% CO_2_ incubator using MEM containing 1% penicillin/streptomycin, 10% FBS. Medium replacement occurred every 1–2 days, and cells were passaged at 48-h intervals [29].

#### 2.9.2. CCK-8 Method

Cell viability was determined via the CCK-8 method [29]. BRL-3a cells were plated in 96-well plates at 2 × 10⁴ cells per well after detachment with 0.25% trypsin when 80–90% confluence was reached. Following 12 h of cell attachment, the culture medium was refreshed, and phenolic acids were diluted to the desired concentrations. Following 48 h of culture, 10 μL of CCK-8 reagent was applied to each well. After 2.5 h, absorbance was recorded at 490 nm, and the corresponding cell viability was determined as follows:

Cell viability (%) = (OD_2_ − OD_1_)/(OD_1_ − OD_0_) × 100, (5)

where OD_0_ is the background, OD_1_ is the blank control, and OD_2_ is the sample-treated group.

#### 2.9.3. Determination of UA Levels in BRL-3A Cells

UA levels were measured following the method of Liu et al. [29]. BRL-3A cells were plated at a density of 5 × 10^5^ cells/well and co-treated with 1 mM xanthine and various concentrations of phenolic acids (FA, CA, GA, and PA), with allopurinol used as a positive control. After 48 h of incubation, supernatants were collected, spun at 1000× *g* for 5 min, then the UA level was quantified with the UA assay kit.

### 2.10. ADMET Computational Simulation

The ADMET properties (absorption, distribution, metabolism, excretion, and toxicity) of FA, CA, GA, and PA were evaluated using computational simulations [30]. These evaluations help the predict the compounds’ in vivo distribution and potential adverse effects. The “Swiss ADME” (http://www.swissadme.ch/index.php), accessed on 15 March 2025, and “pkCSM” (http://biosig.unimelb.edu.au/pkcsm/prediction), accessed on 20 April 2025, platforms were used to model these pharmacokinetic and toxicological parameters.

### 2.11. Statistical Analysis

All results are reported as mean ± SEM based on three to seven independent experiments. Statistical evaluation was performed using PRISM 6 (GraphPad Software, San Diego, CA, USA). Comparisons to the vehicle group were made using one-way ANOVA with Dunnett’s test, and significance was defined as *p* < 0.05.

## 3. Results and Discussion

### 3.1. Antioxidant and XOD Inhibitory Activities of FA, CA, GA, and PA

**Figure 1:**
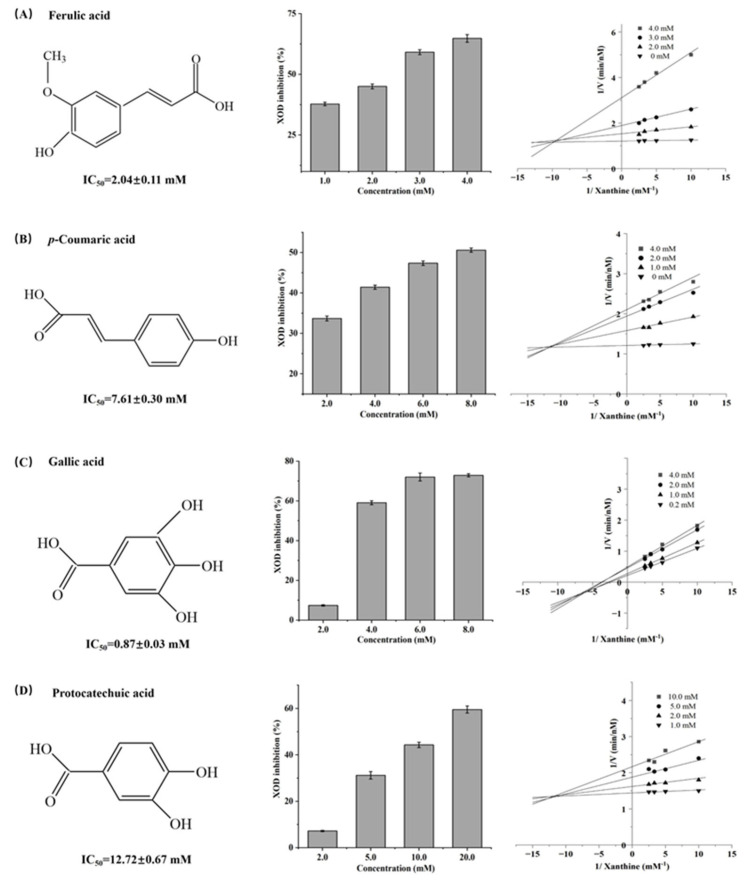
XOD inhibition and corresponding Lineweaver–Burk plots at different concentrations of the four phenolic compounds.

In this study, the antioxidant activities of FA, CA, GA, and PA were evaluated through various methods, including reducing power, ABTS and DPPH radical scavenging capacities, ORAC values, and XOD inhibitory activity. The ORAC value was expressed as the equivalent concentration of the positive control, with higher values indicating stronger antioxidant activity. The other indices were expressed as either the concentration at an absorbance of 0.5 or the 50% inhibitory concentration (IC_50_), with lower values indicating stronger activity. The antioxidant and XOD inhibitory activity data for the four phenolic acids are shown in Table 1 and Figure 1.

The results showed that GA and PA exhibited strong reducing power (0.20 ± 0.01 mM and 0.12 ± 0.01 mM, respectively) and DPPH radical scavenging capacity (0.18 ± 0.01 mM and 0.62 ± 0.00 mM, respectively), outperforming the positive control (Trolox: reducing power 2.62 ± 0.20 mM, DPPH radical scavenging capacity 0.68 ± 0.01 mM) and the other two phenolic acids. This can be attributed to their stronger electron-donor capacity. A significant correlation was observed between reducing power and DPPH radical scavenging capacity, suggesting that both antioxidant mechanisms primarily rely on electron transfer. Furthermore, strong correlations were found between ABTS•^+^-scavenging capacity, ORAC values, and XOD inhibitory activity. Among the phenolic acids, GA showed optimal performance in all three assays, with IC_50_ values of 0.02 ± 0.00 mM for ABTS•^+^-scavenging capacity, 20.12 ± 0.74 mM for ORAC value, and 4.37 ± 0.11 mM for XOD inhibitory activity. In terms of ABTS•^+^-scavenging capacity, ORAC value, and XOD inhibitory activity, FA and CA outperformed PA. The correlation coefficient between XOD inhibitory activity and ABTS•^+^-scavenging capacity was 0.734 (Table S1), indicating that XOD inhibitory activity can be characterized by assessing ABTS•^+^-scavenging capacity.

### 3.2. Types of XOD Inhibition by FA, CA, GA, and PA

The IC_50_ values for the inhibitory activities of FA, CA, GA, and PA on XOD were 2.04 ± 0.11 mM, 7.61 ± 0.30 mM, 0.87 ± 0.03 mM, and 12.72 ± 0.67 mM, respectively (Figure 1). These results indicate that all four phenolic acids inhibit XOD, with FA and GA showing particularly strong activity. This result aligns with the observations reported by Zhao et al., where also reported that FA exhibited stronger XOD inhibition [31]. The inhibition mode was determined via Lineweaver–Burk plots, plotting the inverse of substrate concentration against the inverse of UA production rate (Figure 1). With increasing substrate concentration, the rate of UA production gradually increased. The lines for FA, CA, and PA intersected in the second quadrant, suggesting mixed inhibition with competitive dominance, where GA’s lines intersected in the third quadrant, indicating mixed but predominantly non-competitive inhibition.

The V_max_ and K_m_ values were calculated via nonlinear regression analysis and Michaelis-Menten kinetic modeling (Table S2). For FA, CA, and PA, increasing concentrations led to a reduction in V_max_ accompanied by a rise in K_m_. Based on the kinetic equations for mixed inhibition, the inhibition constant (K_i_) and XOD-substrate complex constant (K_is_) were determined. The K_is_ values for FA (K_i_ = 0.89, K_is_ = 1.78), CA (K_i_ = 7.11, K_is_ = 3.05), and PA (K_i_ = 3.21, K_is_ = 17.76) were significantly higher than the K_i_ values, suggesting stronger binding to XOD than to the XOD-substrate complex. For GA, increasing its concentration decreased both V_max_ and K_m_. This may be due to enhanced binding to the XOD-substrate complex, which increases the binding affinity of the substrate and the enzyme, thereby lowering the K_m_ value. The K_is_ value for GA (3.05) was smaller than the K_i_ value (7.11), indicating that GA binds more strongly to the XOD-substrate complex than to XOD. Lower K value indicate greater inhibitory potency. FA, CA, and PA are all mixed inhibitors, primarily exhibiting competitive inhibition. The K_i_ values follow the order FA (0.89) < CA (1.42) < PA (3.21), indicating that their binding strength to the enzyme decreases in the order of FA > CA > PA. This trend is consistent with their observed inhibitory activity.

### 3.3. Fluorescence Quenching Analysis

**Figure 2:**
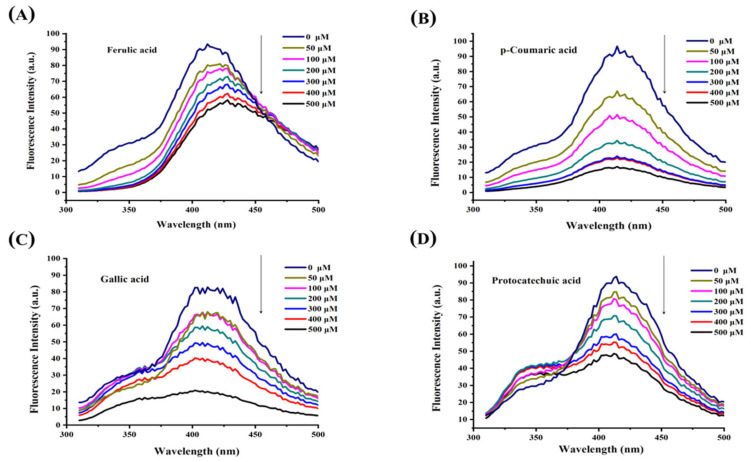
Quenching spectra of XOD fluorescence induced by the four phenolic compounds at different concentrations: (**A**) ferulic acid, (**B**) p-coumaric acid, (**C**) gallic acid, and (**D**) protocatechuic acid.

The binding mechanisms of FA, CA, GA, and PA to XOD were investigated using the fluorescence quenching method at seven different concentrations. Under the experimental fluorescence conditions, XOD exhibited pronounced emission signals at 344 nm and 412 nm. A significant decrease in fluorescence intensity was observed as the phenolic acid concentrations increased, indicating their ability to effectively quench XOD’s intrinsic fluorescence (Figure 2). The Stern–Volmer equation was applied to analyze the fluorescence quenching data [32]:

F_0_/F = 1 + K_sv_ [Q] = 1 + K_q_ τ_0_ [Q], (6)

F_0_/(F_0_ − F) = (1/f_a_) K_a_ [Q] + 1/f_a_, (7)

log [(F_0_ − F)/F] = log K + n log [Q], (8)

F_0_ and F denote the fluorescence intensities of XOD in the absence and presence of quenchers (FA, CA, GA, and PA), respectively. The quenching constant (K_sv_) is, obtained from the linear fitting of F_0_/F against the quencher concentration [Q]. The quenching rate constant is expressed as K_q_, while τ__0__ represents the average lifetime of the fluorophore in the absence of a quencher (typically 10^−9^ s). The effective quenching constant of the accessible fluorophore is designated as K_a_, and f_a_ corresponds to the fraction of fluorescence accessible to quenching. The binding constant is indicated by K, and n stands for the number of binding sites.

The quenching and binding constants are summarized in Table S3, derived from Equations (6)–(8). The K_q_ values of all four phenolic acids exceeded the maximum scattering collisional quenching constant (2 × 10^10^ M^−1^s^−1^), suggesting that fluorescence quenching of XOD is mainly due to static-type quenching via the generation of XOD-phenolic complexes [27]. Analysis of K_sv_ and K values reveals that the stability of the XOD-phenolic complexes decreases in the following order: XOD-CA > XOD-GA > XOD-PA > XOD-FA. The n values, close to 1, suggest a single binding site for all four phenolic acids on XOD [24]. However, the K_a_ values follow a different order: XOD-CA > XOD-FA > XOD-PA > XOD-GA. FA displayed relatively high K_a_ and low K_sv_ values, while GA exhibited the lowest K_a_ and the highest K_sv_ values. These differences may result from phenolic molecules binding to both fluorescent and fluorescent-inactive amino acid residues of XOD. The formation of XOD–phenolic complexes may induce conformational changes in XOD, affecting the microenvironment around fluorescent amino acid residues and leading to changes in fluorescence quenching. Binding to non-fluorescent residues may alter the microenvironment of fluorescent residues, resulting in higher K_sv_ and lower K_a_ values. In contrast, binding to fluorescent residues may not significantly alter XOD’s conformation, resulting in lower K_sv_ and higher K_a_ values.

These results reveal insights into the molecular mechanisms behind the binding interactions between phenolic acids and XOD, offering new perspectives on the color–deficit effect.

### 3.4. CD Spectroscopy

**Figure 3:**
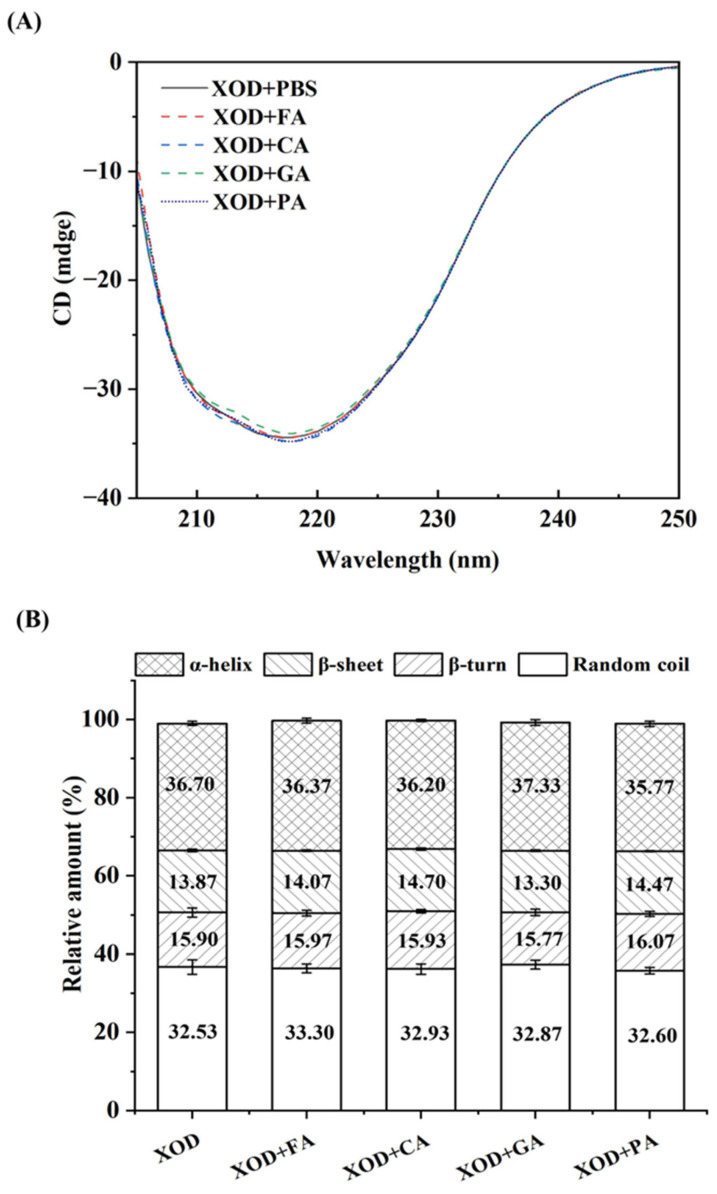
(**A**) Circular dichroism profiles of XOD with or without exposure to four phenolic compounds. (**B**) Evaluation of XOD secondary structural elements from circular dichroism spectra under control and phenolic compound-treated conditions.

CD spectroscopy was employed to detect changes in the secondary structure of XOD. Figure 3A shows the CD spectra of XOD were recorded in the presence of four phenolic acids (FA, CA, GA, and PA) [26]. The results revealed that these phenolic acids induced notable modifications in the CD spectra of XOD, particularly in the 208–224 nm range. α-helical structures typically show characteristic negative peaks at 208 nm and 222 nm, while β-sheet structures show negative peaks around 215 nm. These findings indicated that all four phenolic acids altered the α-helical and β-sheet structures of XOD, enhancing its rigidity. This rigidity may restrict the formation of the XOD active site and reduce its ability to bind substrates effectively.

The CONTIN program was used to determine the secondary structure composition of XOD (Figure 3B) [25]. The data showed that the phenolic acids significantly affected the proportions of α-helix, β-sheet, β-turn, and random structures in XOD. Specifically, the α-helix content of XOD decreased from 36.70% to 36.37%, 36.20%, and 35.77% after adding FA, CA, and PA, respectively. In contrast, GA increased the α-helix content to 37.33%. As for the β-sheet structure, FA, CA, and PA significantly increased the β-sheet content, while GA reduced it to 13.30%. The β-sheet content increased to 14.07% (FA), 14.70% (CA), and 14.47% (PA). Notably, GA had the most pronounced effect on both α-helical and β-sheet structures of XOD, increasing rigidity and altering its function. These results indicate that GA has a more pronounced effect on altering the secondary structure of XOD and inhibiting its enzymatic activity.

In conclusion, all four phenolic acids induced unfolding and rearrangement of XOD’s secondary structure. The differences in the extent of these structural changes likely contribute to the varying inhibitory activities of each phenolic acid against XOD.

### 3.5. Molecular Docking

**Figure 4:**
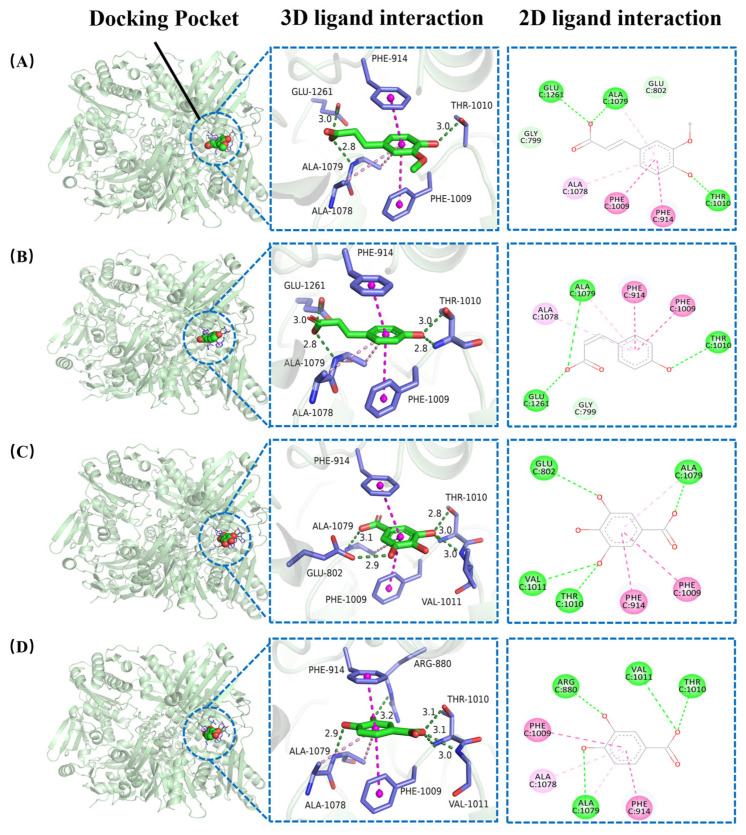
Molecular docking results showing the interactions between XOD and four phenolic acids: (**A**) ferulic acid, (**B**) p-coumaric acid, (**C**) gallic acid, and (**D**) protocatechuic acid.

Molecular docking is a key technique in bioinformatics and computational chemistry, primarily used to study interactions between small-molecule compounds and proteins. It provides insights into binding sites, binding energies, and noncovalent interactions [26]. Figure 4 and Table S4 show the binding sites of FA, CA, GA, and PA with XOD. The results indicate that all four phenolic acids bind to the molybdenum pterin structural domain of XOD, with binding sites located deep within the active pocket. This suggests they effectively inhibit substrate binding to XOD. The interactions of these phenolic acids with XOD’s amino acid residues were further analyzed through three-dimensional and two-dimensional interaction analysis. FA and CA exhibited similar mechanisms, where their polar functional groups formed hydrogen bonds with Ala1079, Glu1261, and Thr1010 of XOD. The benzene rings of these compounds engaged in π-π stacking interactions with Phe914 and Phe1009, as well as π-alkyl interactions with Ala1078. The polar functional group of GA forms hydrogen bonds with Ala1079, Glu802, Thr1010, and Val1011 of XOD, while its benzene ring forms π-π stacking with Phe914 and Phe1009, and π-alkyl interactions with Ala1079. The polar functional groups of PA interacted with Ala1079, Arg880, Thr1010, and Val1011 of XOD through hydrogen bonds, while its benzene ring engaged in π-π stacking with Phe914 and Phe1009, and π-alkyl interactions with Ala1078 and Ala1079.

Several amino acid residues in the XOD catalytic center are crucial for the catalytic reaction, such as Arg880, Glu802, and Glu1261. These residues are directly involved in the substrate conversion [33]. Phe914, Phe1009, and Val1011 at the entrance of the active center regulate substrate or inhibitor entry by regulating the pore size or the interacting with the substrate. These residues are key factors influencing the rate of the XOD catalytic reaction and substrate entry [33]. Molecular docking analysis further revealed that the four phenolic acids inhibit XOD’s catalytic activity via hydrogen bonding and conjugative contacts with these critical residues.

### 3.6. Molecular Dynamics Simulations

**Figure 5:**
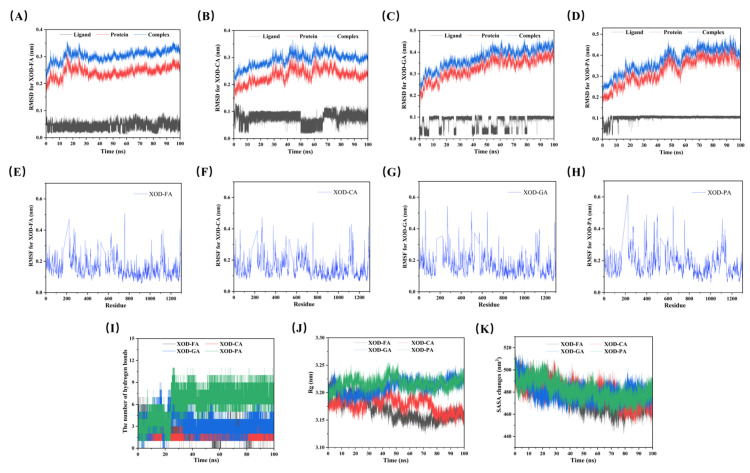
Molecular dynamics simulations conducted over 100 ns, Figures (**A**–**D**) show the root-mean-square deviation (RMSD) of the XOD-FA, XOD-CA, XOD-GA, and XOD-PA systems; Figures (**E**–**H**) display the root-mean-square fluctuation (RMSF) of these systems. Figure (**I**) illustrates the variation in hydrogen bond numbers; Figure (**J**) shows the change in radius of gyration (Rg); and Figure (**K**) depicts the variation in sol-vent-accessible surface area (SASA).

Molecular dynamics simulations were performed to investigate the influence of four phenolic acids on the conformational dynamics and stability of XOD. The structural stability of each XOD-phenolic acid complex was monitored using RMSD. As illustrated in Figure 5A–D, all XOD-phenolic acid complexes reached equilibrium between 25–70 ns, with RMSD values stabilizing around 0.3 to 0.5 nm. Compared with XOD alone, the RMSD values of the complexes were 0.04–0.07 nm higher, indicating that the phenolic acids influenced the initial conformation of XOD. At 100 ns, the RMSD values were 0.3157 nm (XOD-FA), 0.2929 nm (XOD-CA), 0.4213 nm (XOD-GA), and 0.4695 nm (XOD-PA). Among them, XOD-FA and XOD-CA exhibited better stability, with lower RMSD values. Notably, the RMSD of CA and GA fluctuated more in their respective complexes, suggesting these two phenolic acids interacted more dynamically with XOD.

The impact of the phenolic acids on the fluctuations of XOD residues was analyzed using RMSF. As shown in Figure 5E–H, the phenolic acids exhibited similar effects on RMSF values in five specific regions of XOD: the α-helix (110–150), β-sheet (740–810), random coil structure (900–920), pocket region (1020–1085), and flexible side chains (1265–1290). This suggests that the phenolic acids primarily alter the α-helix, β-sheet, and random coil structures of XOD, consistent with the findings of Pan et al. [34]. The distinct phenolic acids influenced XOD’s flexibility by influencing different secondary structure regions. Lower average RMSF values corresponded to greater residue stability, with the XOD-FA and XOD-CA complexes exhibiting higher stability compared to XOD-GA and XOD-PA.

GA and PA formed more hydrogen bonds with XOD, which contributed to the stabilization of their complexes (Figure 5I). Conversely, the Rg of the XOD-FA and XOD-CA complexes was smaller, suggesting their structures were more compact. As illustrated in Figure 5J, the Rg values for XOD-FA and XOD-CA were approximately 3.12–3.18 nm, lower than those of the other complexes, suggesting that FA and CA facilitated protein folding or induced specific conformational adjustments. SASA analysis revealed that the surface area of XOD decreased upon binding with phenolic acids (Figure 5K). Specifically, the XOD-FA and XOD-CA complexes exhibited a greater reduction in surface area. This was likely due to their smaller Rg and more compact structure, which contributed to their higher stability.

### 3.7. UA-Lowering Activity of Phenolic Acids in BRL-3A Cells

**Figure 6:**
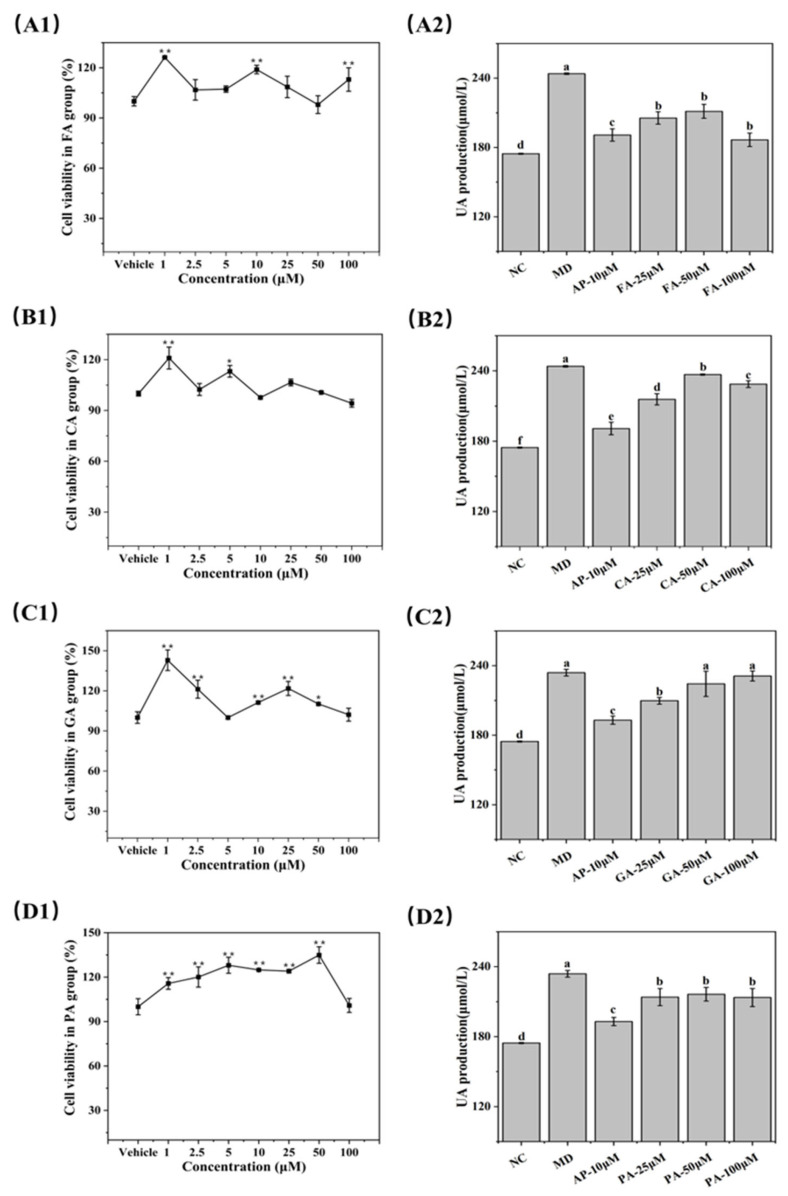
Assessment of BRL-3A cells after treatment with four phenolic compounds: FA (**A**), CA (**B**), GA (**C**), and PA (**D**). Cell viability is shown in (**A1**–**D1**), and uric acid levels are presented in (**A2**–**D2**). Groups not sharing the same letter (e.g., a, b, c) are significantly different (*p* < 0.05). * Significant correlations at *p* < 0.05. ** Significant correlations at *p* < 0.01.

The effects of four phenolic acids on BRL-3A cells’ viability were evaluated using the CCK-8 assay. After 48 h of exposure to various concentrations of the phenolic acids, cell proliferation rates were assessed, as shown in Figure 6(A1–D1). At concentrations ranging from 1 to 100 μM, the phenolic acids increased cell viability under certain conditions, but did not significantly affect BRL-3A cell survival. Therefore, the highest concentration of 100 μM was considered non-toxic to BRL-3A cells.

To model high UA levels, BRL-3A cells were treated with xanthine for 48 h. As shown in Figure 6(A2–D2)**,** the UA level in the model group was significantly higher than that in the normal control (NC) group (*p* < 0.05). The positive control group (allopurinol, AP) showed significantly lower UA levels compared with the model group (*p* < 0.05). UA levels were reduced to varying degrees following treatment with different concentrations of phenolic acids. FA, CA, and PA significantly reduced UA levels in the 25–100 μM range (*p* < 0.05), while GA only significantly reduced UA levels at 25 μM. These results suggest that the four phenolic acids may help mitigate xanthine-induced high UA levels in BRL-3A cells.

Overall, FA, CA, and PA significantly reduced UA levels at concentrations from 25 to 100 μM. Since UA levels are influenced by various factors, further investigations are required to elucidate the mechanisms responsible for the UA-lowering effects of these phenolic acids.

### 3.8. Physicochemical and ADMET Performance Prediction

The SMILES formats of the four phenolic acids were uploaded to the SwissADME website for calculation. The results showed that all four compounds complied with Lipinski’s five rules, which include molecular weight (Mw) < 500 Da, hydrogen bond acceptor (H-Ac) ≤ 10, hydrogen bond donor (H-Do) ≤ 5, octanol-water partition coefficient (Log P) ≤ 5, and rotatable bonds (NRBs) ≤ 10. These findings support their potential as orally active substances (Table 2). According to Cerqueira et al. [35], the polar surface area (TPSA) should range from 20 Å to 140 Å for optimal drug absorption and distribution. The TPSA values of the four phenolic acids ranged from 57.53 to 97.99 Å, indicating favorable bioavailability. GA exhibited the highest TPSA value (97.99 Å), suggesting stronger receptor interactions. Solubility is a key factor influencing absorption. Values below −10 indicate insolubility, −10 to −6 weak solubility, −6 to −2 partial solubility, −2 to 0 high solubility, and values above 0 extreme solubility [36]. The solubility values of the four phenolic acids ranged from −2.11 to −1.64, indicating good solubility. ADMET analysis revealed that three phenolic acids (except GA) had high intestinal absorption probabilities (77.579–93.494%), while GA had a significantly lower absorption rate (43.374%). Caco-2 permeability analysis showed that FA, CA, and PA had high permeability, whereas GA exhibited poor permeability (−0.081 cm/s). All four compounds had blood–brain barrier permeability values below 0.3, suggesting limited ability to cross the barrier. Metabolic analysis indicated that none of the four phenolic acids were substrates for CYP2D6 or CYP3A4, suggesting high metabolic stability. pKCSM predictions further showed that they are not OCT2 substrates, implying no reliance on OCT2 transport for renal clearance. Toxicity assessment revealed no hepatotoxicity for any of the four phenolic acids.

In conclusion, the four phenolic acids complied with Lipinski’s five rules, demonstrating good oral activity, bioavailability, and solubility. They exhibited excellent intestinal absorption, high Caco-2 permeability (except GA), metabolic stability, and no OCT2 transport dependency. Additionally, none of them showed hepatotoxicity, indicating potential for development as functional food ingredients.

### 3.9. Physicochemical and ADMET Performance Prediction Structure-Activity Relationship of XOD Inhibition by Phenolic Acids

In the preliminary structure-activity relationship (SAR) study, the inhibition of XOD by four phenolic acids was evaluated by assessing their IC_50_ values of XOD inhibition. The results illustrated that the methoxy group at C3 of FA plays a critical role in inhibiting XOD compared to CA. Additionally, the inhibitory efficacy of GA was approximately 14.62 fold greater than PA, suggesting that the hydroxyl group at C5 of GA significantly enhances its XOD inhibitory ability. Liu et al. also reported that substituents on the benzene ring in phenolic acids notably increase their inhibitory effect on XOD [37]. All four phenolic acids acted as mixed-type inhibitors of XOD, interacting with both the free enzyme and the enzyme–substrate complex. This binding alters the microenvironment of tryptophan (Trp) and tyrosine (Tyr), promoting rearrangement of the XOD secondary structure, which affects its catalytic activity and hydrophobicity. Docking studies and kinetic simulations suggested that the strong binding of phenolic acids to XOD is mediated by hydrogen bonding and π-π conjugation.

Furthermore, the UA-lowering effects of the four phenolic acids were assessed using a xanthine-induced BRL-3A cell model of hyperuricemia. At 25 μM, all phenolic acids significantly reduced intracellular UA concentration, although no dose-dependent relationship was observed. Despite GA’s high activity in the chemical assay, its activity in the cellular model was lower. This discrepancy may be attributed to GA’s relatively low intestinal absorption and large polar surface area, which could hinder its cellular uptake.

Phenolic acids are associated with UA synthesis, oxidative stress, and inflammation. it is hypothesized that phenolic acids may alleviate hyperuricemia by reducing UA production, oxidative stress and inflammation. However, one limitation of the present study is the absence of in vivo evidence. In particular, the specific effects of the four phenolic acids on hyperuricemia and their underlying mechanisms have not yet been investigated in animal models or clinical settings. Future research should focus on in vivo experiments to address these gaps and provide a clearer understanding of the therapeutic potential and health benefits of these compounds.

## 4. Conclusions

The inhibitory mechanism of four phenolic acids (FA, CA, GA, and PA) against XOD was systematically investigated. All compounds exhibited mixed-type inhibition. Intrinsic fluorescence of XOD was effectively quenched through a static mechanism, suggesting notable alterations in its hydrophobic environment and secondary structure. Docking and dynamics analyses indicated that phenolic acids bound at the active site via hydrophobic and hydrogen bonding, thereby hindering substrate binding and inducing structural rearrangements. Among them, FA and CA formed more stable complexes with XOD, as indicated by lower Rg, RMSD, and RMSF values. Cellular assays demonstrated a reduction in UA levels upon treatment with the phenolic acids. ADMET analysis suggested favorable safety and absorption profiles, highlighting their potential as candidates for further development. These findings provide theoretical support for phenolic acid-based XOD inhibitors.in hyperuricemia management.

## Figures and Tables

**Table 1:** Antioxidant activities of four phenolic acids. Groups not sharing the same letter (e.g., a, b, c) are significantly different (*p* < 0.05).

Compound	Reducing Power (mM)	IC50 Values of ABTS Assay (mM)	IC50 Values of DPPH Assay (mM)	ORAC Value (mmol TE/mmol)
Ferulic acid	0.74 ± 0.05 c	0.30 ± 0.00 c	0.80 ± 0.03 b	7.99 ± 0.24 c
p-Coumaric acid	6.98 ± 0.13 a	0.23 ± 0.01 c	19.39 ± 1.24 a	9.64 ± 0.27 b
Gallic acid	0.20 ± 0.01 d	0.02 ± 0.00 d	0.18 ± 0.01 b	20.12 ± 0.74 a
Protocatechuic acid	0.12 ± 0.01 d	0.40 ± 0.02 b	0.62 ± 0.00 b	6.63 ± 0.09 d
Positive control	2.62 ± 0.20 b (Trolox)	3.41 ± 0.12 a (Trolox)	0.68 ± 0.01 b (Trolox)	

**Table 2:** Computationally predicted physicochemical characteristics, pharmacokinetics, and toxicity profiles of the four phenolic acids.

Compound	Ferulic Acid	p-Coumaric Acid	Gallic Acid
Mw	194.18	164.16	170.12
H-Ac	4	3	5
H-Do	2	2	4
Log P	1.25	1.43	0.59
NRB	3	2	1
TPSA	66.76	57.53	97.99
Water solubility (log mol/L)	−2.816	−2.378	−2.56
HIA (%)	77.579	93.494	43.374
Caco-2 permeability (log Papp, cm/s)	1.757	1.21	−0.081
BBB permeability (log BB)	0.175	−0.225	−1.102
CYP2D6	No	No	No
CYP3A4	No	No	No
Renal OCT2 substrate	No	No	No
Hepatotoxicity	No	No	No

MW, molecular weight; H-Ac, hydrogen bond acceptor count; H-Do, hydrogen bond donor count; Log P, calculated octanol/water partition coefficient; NRB, rotatable bond number; TPSA, topological polar surface area; HIA, predicted human intestinal absorption; BBB, blood–brain barrier permeability.

## Data Availability

The data are contained within the article and Supplementary Materials. Other original data supporting reported results are available upon request.

## Supplementary Materials

The following supporting information can be downloaded at: https://www.mdpi.com/article/10.3390/foods14193404/s1, Figure S1: Optimal 2D model of docking between sixteen ASE peptides and ABTS radical; Figure S2: Optimal 2D model of docking between sixteen ASE peptides and DPPH radical; Figure S3: Active-site analysis of the sixteen ASE peptides by HOMO and Mulliken charge distribution, and bond length. Table S1: Antioxidant activities of the sixteen ASE peptides. Data with different letters in the same test indicate significant differences *(p* < 0.05).; Table S2: Correlation study of chemical antioxidant activity with various indices in erythrocyte models. * Significant correlations at *p* < 0.05. ** Significant correlations at *p* < 0.01.
