# Supplementary material for: Inhibition of Xanthine Oxidase by Four Phenolic Acids: Kinetic, Spectroscopic, Molecular Simulation, and Cellular Insights
Source: Foods. 2025 Oct 1;14(19):3404. doi: 10.3390/foods14193404 (PMC12523838; doi:10.3390/foods14193404)
Supplement: Supplementary file 1 [file foods-14-03404-s001.zip › foods-3841581-supplementary.pdf]

Inhibition of xanthine oxidase by four phenolic acids: Kinetic, spectroscopic, molecular simulation, and cellular insights

Xiao Wang [a*], Di Su [c*], Xinyu Luo [a], Bingjie Chen [a], Khushwant S. Bhullar [d], Hongru Liu [a], Chunfang Wang[a], Jinglin Zhang [a], Longshen Wang [a], Hang Yang [b*],Wenzong Zhou[b*]

[a] *Crop Breeding and Cultivation Research Institution, Research Center for Agricultural Products Preservation and Processing, Shanghai Academy of Agricultural Sciences, Shanghai, China*

[b] *Key Laboratory of Integrated Rice-Fish Farming Ecosystem,Ministry of Agriculture and Rural Affairs, Shanghai Academy of Agricultural Sciences, Shanghai, China*

[c] *Teaching Experimental Center for Pharmacy, School of Pharmaceutical Sciences, Shanghai Jiao Tong University, 800 Dongchuan Road, Shanghai, 200240, P. R. China.*

[d] *Department of Agricultural Food & Nutritional Science, University of Alberta, Edmonton T6G 2P5, Canada*

[*] Corresponding author, Email: yanghangqu@foxmail.com; hrdxwandg@gmail.com

Table S1 An Correlation analysis of antioxidant and XOD inhibitory activities of four phenolic acids.

Parameters	Reducing power (mM)	IC_{50} values of ABTS assay (mM)	IC_{50} values of DPPH assay (mM)	ORAC value (mmol TE/mmol)	IC_{50} values of XOD inhibitory activities (mM)
Reducing power (mM)	1				
IC_{50} values of ABTS assay (mM)	-0.018	1			
IC_{50} values of DPPH assay (mM)	.997**	-0.006	1		
ORAC value (mmol TE/mmol)	-0.182	-.964**	-0.181	1	

IC_{50} values of XOD inhibitory activities (mM)	0.183	.734**	0.234	-0.655*	1

* Significant correlations at $p < 0.05$. ** Significant correlations at $p < 0.01$.

Table S2 Inhibition kinetics parameters and types of four phenolic acids to XOD

**Compound**	**Concentrations (mM)**	**K_i (mM)**	**K_{is} (mM)**	**V_{max} (mM/min)**	**K_m (10^{-3} mM)**	**Inhibition types**
Ferulic acid -7.0	0.0	0.89	1.78	1.42	13.04	Mixed inhibition
	2.0			1.39	18.43	
	3.0			1.04	23.51	
	4.0			0.95	37.65	
p-Coumaric acid -7.1	0.0	1.42	6.04	0.83	14.66	Mixed inhibition
	1.0			0.79	16.10	
	2.0			0.74	16.66	
	4.0			0.68	18.95	
Gallic acid -6.5	0.2	7.11	3.05	4.90	417.11	Mixed inhibition
	1.0			4.83	422.86	
	2.0			4.72	406.67	
	4.0			3.61	347.59	
Protocatechuic acid -6.5	1.0	3.21	17.76	0.79	9.62	Mixed inhibition
	2.0			0.72	12.12	
	5.0			0.69	12.38	
	10.0			0.68	15.33	

Table S3 The quenching constants (K_{sv}), quenching rate constants (K_q), effective quenching constants (K_a), binding constants (K), and number of binding sites (n) for the interaction of four phenolic compounds with XOD at 298 K.*

**Compounds**	**K_{sv} (10^4 L*mol^{-1})**	**K_q (10^{12} L*mol^{-1}*s^{-1})**	**r^a**	**K_a (10^4 L*mol^{-1})**	**r^b**	**K (10^3 L*mol^{-1})**	**n**	**r^c**
Ferulic acid	0.16 ± 0.01^d	0.16 ± 0.01^d	0.9991	0.66 ± 0.02^b	0.9951	0.14 ± 0.00^d	0.70 ± 0.01^d	0.9911
p-Coumaric acid	0.91 ± 0.01^a	0.91 ± 0.01^a	0.9953	0.87 ± 0.01^a	0.9990	10.01 ± 0.45^a	1.01 ± 0.00^b	0.9927
Gallic acid	0.26 ± 0.01^b	0.26 ± 0.01^b	0.9971	0.20 ± 0.01^d	0.9910	7.18 ± 0.18^b	1.13 ± 0.00^a	0.9936
Protocatechuic acid	0.18 ± 0.01^c	0.18 ± 0.01^c	0.9997	0.29 ± 0.01^c	0.9962	1.39 ± 0.10^c	0.98 ± 0.02^c	0.9954

r^a, r^b, r^c are the correlation coefficients for the K_{sv}, K_q, K_a and K values, respectively. when the letters followed by the values are completely different, the values in the same column were significantly different ($p < 0.05$). Otherwise, the values in the same column were not significantly different ($p > 0.05$)

Table S4 Analysis of the binding affinities of the four phenolic acids with XOD.

**Compounds**	**Binding affinity (kcal/mol)**	**Phenolic acid-amino acid interactions**	
		**Hydrogen bond interactions (Å)**	**Hydrophobic bond interactions**
Ferulic acid	-6.9	Ala1079 (2.8), Glu1261 (3.0), Thr1010 (2.8), Thr1010 (3.0)	Ala1078, Ala1079, Phe 914, Phe 1009
p-Coumaric acid	-7.1	Ala1079 (2.8), Glu1261 (3.0), Thr1010 (2.8), Thr1010 (3.0)	Ala1078, Ala1079, Phe 914, Phe 1009
Gallic acid	-6.5	Ala1079 (3.1), Glu802 (2.9), Thr1010 (2.8), Thr1010 (3.0),Val1011 (3.0)	Ala1079, Phe 914, Phe 1009
Protocatechuic acid	-6.5	Ala1079 (2.9), Arg880 (3.2),Thr1010 (3.1), Thr1010 (3.1),Val1011(3.0)	Ala1078, Ala1079, Phe 914, Phe 1009
